# Periodontal Intervention in Soft Tissue-Embedded Orthodontic Appliances: A Multidisciplinary Healing Approach and Case Series

**DOI:** 10.7759/cureus.113207

**Published:** 2026-07-23

**Authors:** Disha Gupta, Akash Bhatnagar, Zoya Chowdhary

**Affiliations:** 1 Periodontology, Teerthanker Mahaveer Dental College and Research Centre, Moradabad, IND; 2 Pediatric and Preventive Dentistry, Teerthanker Mahaveer Dental College and Research Centre, Moradabad, IND; 3 Dentistry, Government District Hospital, Reasi, IND

**Keywords:** mucosal overgrowth, orthodontic mini-implants, orthodontics, periodontics, transpalatal arch

## Abstract

Embedding of orthodontic appliances within the oral mucosa represents an infrequent but clinically relevant complication that may result in pain, mucosal injury, and functional discomfort if not identified early. This report describes two cases managed in the Department of Periodontology. The first case involved a 17-year-old female presenting with a submerged orthodontic mini-implant in the maxillary right second premolar region associated with pain during mastication and speech. The second case involved a 22-year-old female with partial mucosal embedding of the U-shaped loop of a transpalatal arch in the palatal tissue. In both patients, the soft tissue-embedded appliances were surgically retrieved under local anesthesia, followed by satisfactory healing within 10-15 days. These cases highlight the importance of periodic monitoring of orthodontic appliances and demonstrate the critical role of the periodontist in the multidisciplinary management of orthodontic complications.

## Introduction

Temporary anchorage devices (TADs) and palatal appliances such as transpalatal arches (TPAs) have become integral components of contemporary orthodontic therapy by enabling effective anchorage control during complex tooth movement. Although these appliances demonstrate high clinical success rates, their use may be associated with biological complications including soft-tissue inflammation, mucosal hypertrophy, peri-implant irritation, and, in rare situations, complete mucosal embedding [1,2]. The risk of such complications is influenced by factors including appliance design, insertion depth, oral hygiene status, follow-up compliance, and the nature of the surrounding soft tissue. Previous reports have shown that appliances placed in non-keratinized mucosa are more susceptible to inflammatory tissue overgrowth and subsequent failure compared with those positioned within keratinized tissue [3].

Soft tissue (mucosal) embedding of orthodontic components represents an uncommon iatrogenic complication that may develop gradually due to chronic irritation and progressive soft-tissue proliferation over the appliance surface [4,5]. Patients frequently present with pain during mastication or speech, localized inflammation, difficulty in appliance maintenance, and psychological discomfort. In these situations, routine chairside removal may not be feasible, and surgical exposure becomes necessary to facilitate safe retrieval while minimizing trauma to adjacent periodontal structures [6,7].

This case series reports two unusual soft-tissue complications encountered during orthodontic treatment involving fixed appliances. One case presented with complete mucosal coverage of an orthodontic mini-implant, whereas the other involved partial embedding of the loop of a TPA due to soft-tissue overgrowth. Both complications were treated successfully by surgical retrieval under local anesthesia using a conservative scalpel-assisted approach, with uneventful healing and no postoperative complications. Although such events are uncommon, they can cause patient discomfort and interfere with ongoing orthodontic treatment if left unrecognized. These cases highlight the value of regular follow-up visits, careful evaluation of appliance-tissue relationships, and timely surgical management in achieving predictable clinical outcomes and preventing further complications. This work has not been previously presented in part or in full at any conference, symposium, or meeting.

## Case presentation

Case 1

**Figure 1:**
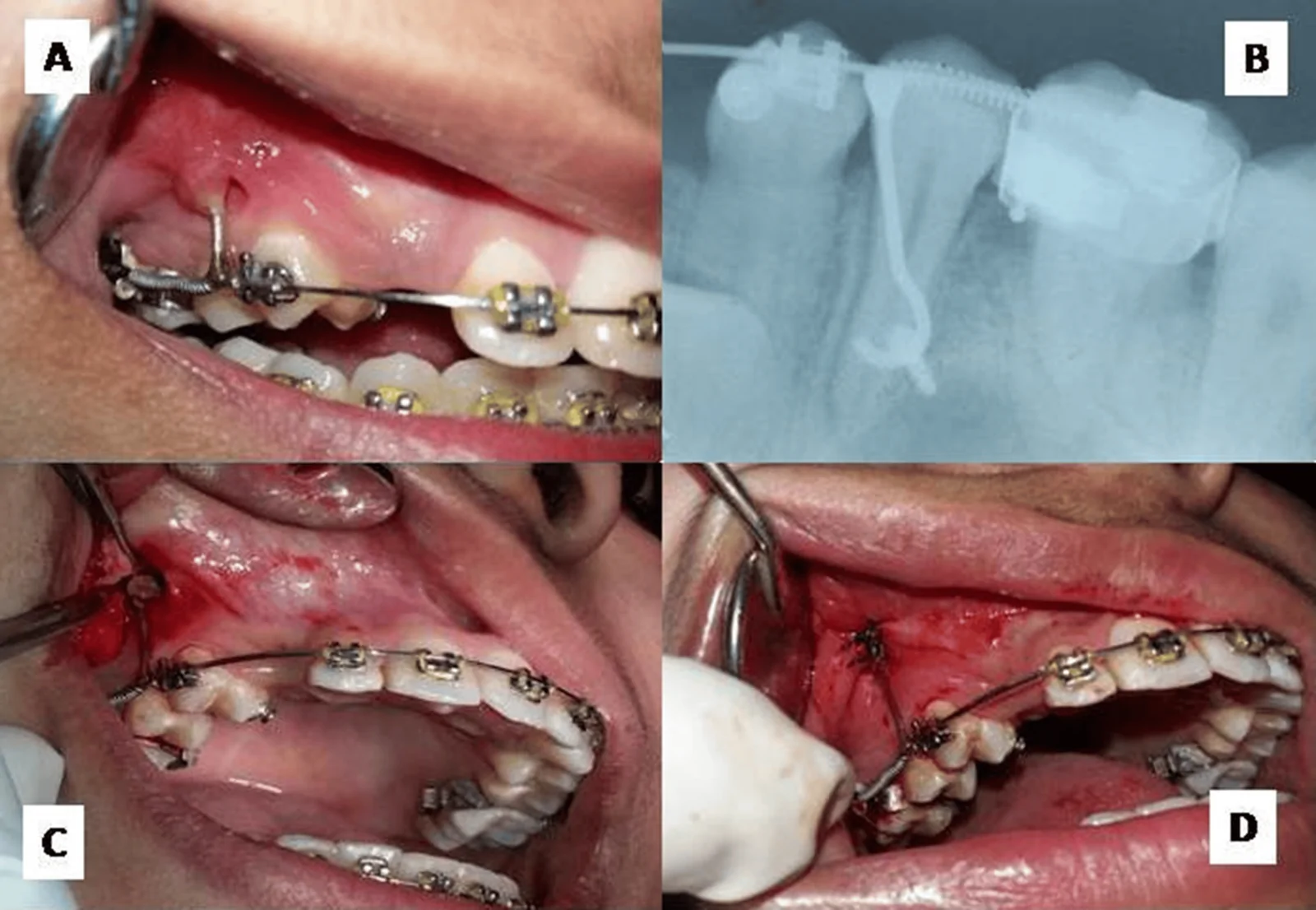
Management of a submerged orthodontic mini-implant. (A) Preoperative clinical view showing inflamed overgrown buccal mucosa. (B) Intraoral periapical radiograph showing the mini-implant without evidence of bony changes. (C) Surgical exposure of the submerged mini-implant after flap elevation. (D) Wound closure with 4-0 black silk sutures.

A 17-year-old female patient was referred from the Department of Orthodontics to the Department of Periodontology with a chief complaint of pain in the right maxillary posterior region. The patient reported discomfort during mastication, speech, and mouth movements. Clinical examination revealed erythematous and inflamed alveolar mucosa in relation to the maxillary right second premolar region (Figure 1, Panel A). The patient’s oral hygiene status was fair.

Dental history revealed placement of an orthodontic mini-implant (FORESTADENT Bernhard Förster GmbH, Pforzheim, Germany) in the same region three months earlier. An intraoral periapical radiograph confirmed the presence of a mini-implant adjacent to the root of the maxillary right second premolar (Figure 1, Panel B). Clinical and radiographic findings suggested soft-tissue overgrowth resulting in complete mucosal coverage of the mini-implant, leading to localized inflammation and pain.

Following informed consent, surgical exposure of the submerged mini-implant was performed under local anesthesia using 2% lignocaine hydrochloride with 1:80,000 adrenaline (Lignospan Special, Septodont, Saint-Maur-des-Fossés, France). A mucoperiosteal flap was raised using a No. 12 Bard-Parker blade (Western Surgical, Ahmedabad, Gujarat, India), exposing the embedded mini-implant (Figure 1, Panel C). Granulation tissue surrounding the implant was debrided, and the surgical site was irrigated with sterile saline. The flap was repositioned and secured using 4-0 silk sutures (Ethicon, Johnson & Johnson Private Limited, Mumbai, India) (Figure 1, Panel D).

Postoperatively, the patient was prescribed diclofenac sodium (50 mg) with serratiopeptidase (15 mg) every eight hours for three days, amoxicillin 500 mg three times daily for five days, and 0.2% chlorhexidine mouthwash (Hexidine, ICPA Health Products Ltd., Mumbai, India) twice daily.

**Figure 2:**
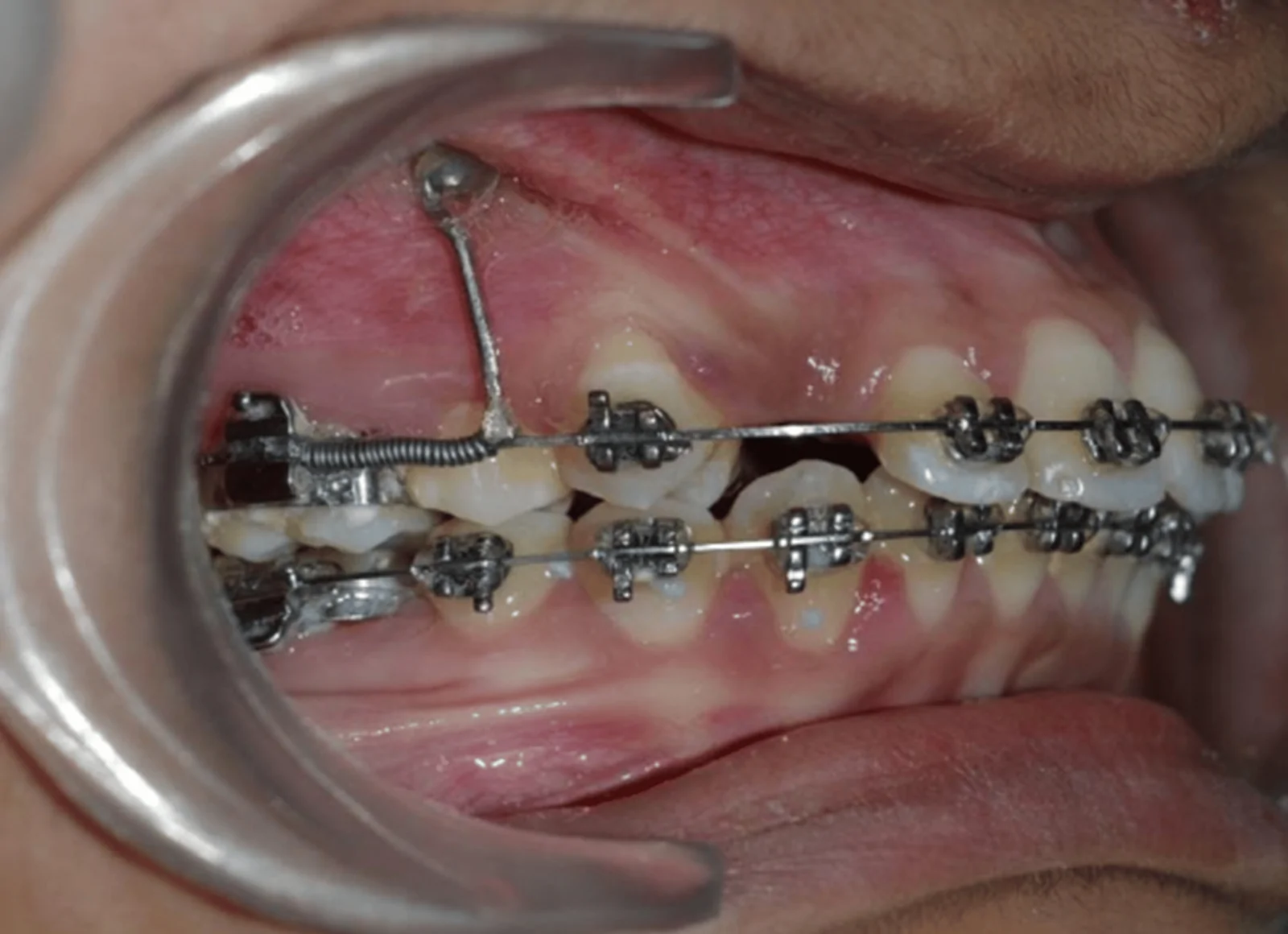
Postoperative clinical view at 15 days showing satisfactory healing around the orthodontic mini-implant.

At the 15-day follow-up visit, clinical examination demonstrated satisfactory healing with complete resolution of inflammation and symptoms (Figure 2). The patient reported no discomfort and was subsequently referred back to the Department of Orthodontics for continuation of orthodontic treatment.

Case 2

**Figure 3:**
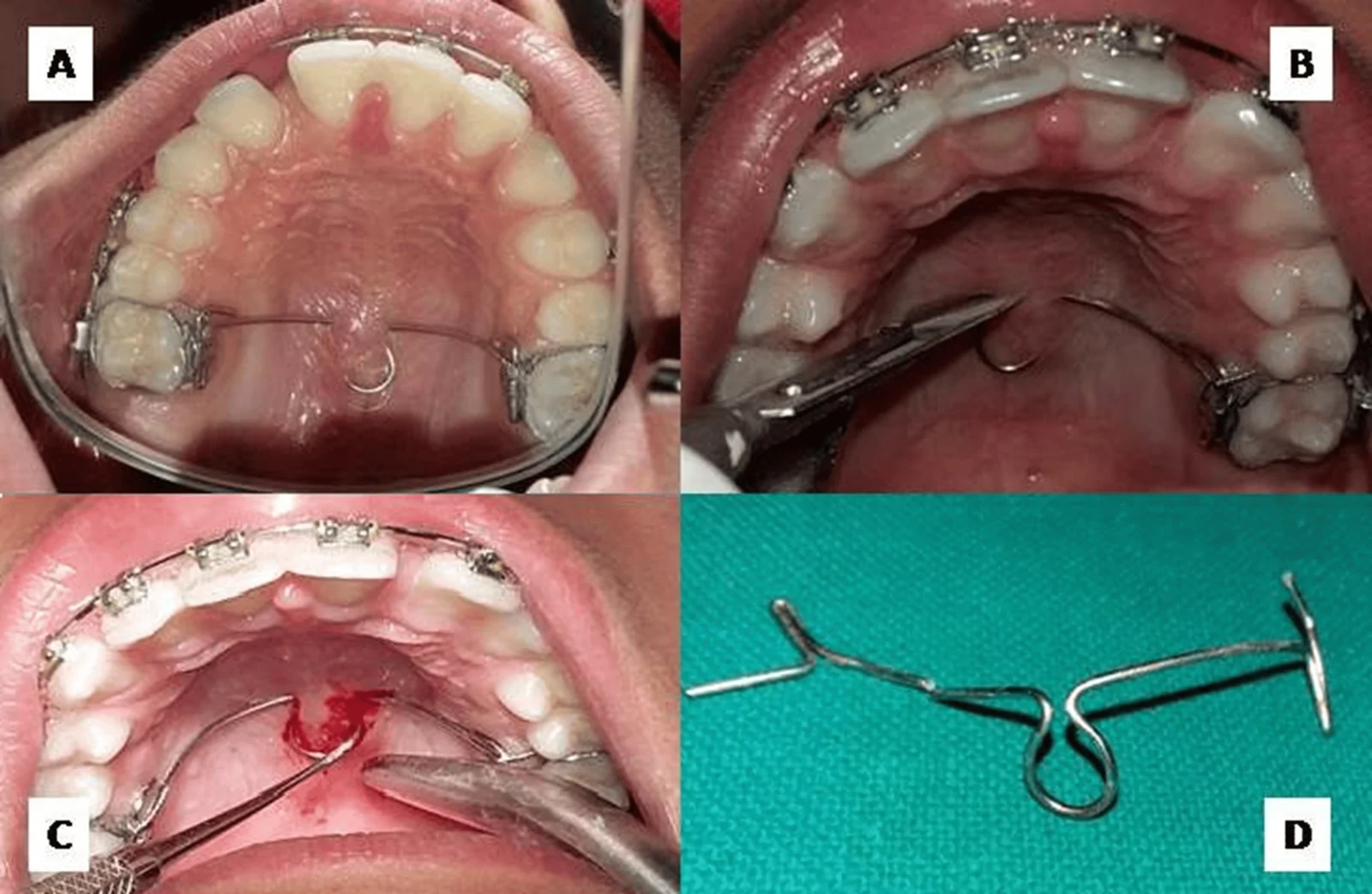
Surgical retrieval of an embedded transpalatal arch. (A) Preoperative clinical view showing embedding of the U-shaped loop of the transpalatal arch within the palatal mucosa. (B) Incision made in the palatal mucosa to expose the embedded loop. (C) Retrieval of the embedded portion of the appliance using the blunt end of a periosteal elevator. (D) Removal of the transpalatal arch appliance following complete exposure.

A 22-year-old female patient was referred from the Department of Orthodontics to the Department of Periodontology for removal of a TPA. Intraoral examination revealed partial embedding of the U-shaped loop of the TPA (Round stainless steel wire; Metro Orthodontics, Hyderabad, Telangana, India) within the palatal mucosa (Figure 3, Panel A). Mild inflammation of the surrounding soft tissue was evident, and palpation confirmed the presence of the embedded loop beneath the mucosal surface.

Based on the clinical findings, surgical retrieval of the embedded TPA was planned. Written informed consent was obtained before the procedure. Local anesthesia was administered using 2% lignocaine hydrochloride with 1:80,000 adrenaline (Lignospan Special, Septodont, Saint-Maur-des-Fossés, France). A releasing incision was made around the margins of the embedded TPA loop using a No. 11 Bard-Parker blade (Western Surgical, Ahmedabad, Gujarat, India) (Figure 3, Panel B). The embedded segment of the appliance was carefully exposed and retrieved with minimal trauma to the surrounding tissues (Figure 3, Panel C).

**Figure 4:**
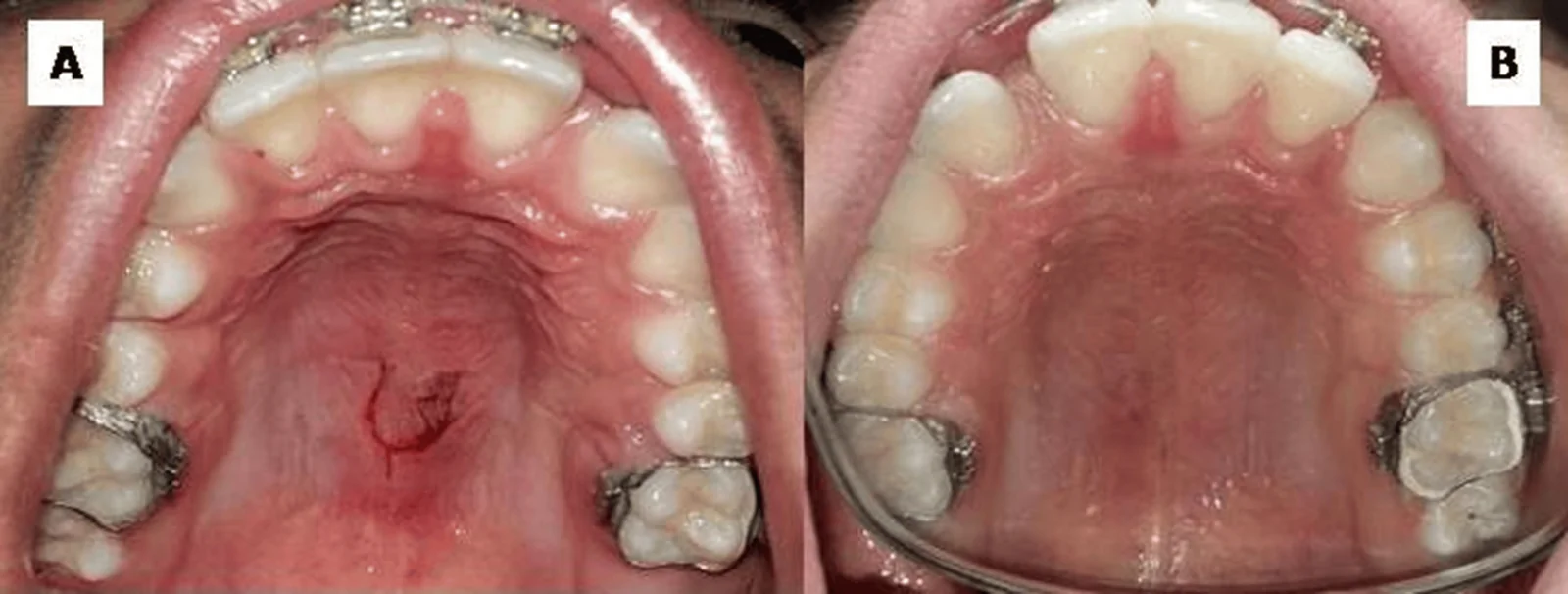
Postoperative views following removal of the embedded transpalatal arch. (A) Immediate postoperative clinical view. (B) Ten-day follow-up showing complete soft-tissue healing.

Following exposure, the TPA was sectioned into two halves to facilitate atraumatic removal from the palatal tubes of the orthodontic bands (Ormco, Brea, California, USA) (Figure 3, Panel D). After complete removal of the appliance, the surgical site was irrigated with sterile saline, and hemostasis was achieved using gentle pressure with sterile gauze (Figure 4, Panel A). The wound was left to heal by secondary intention.

Postoperative instructions were provided, and the patient was prescribed diclofenac sodium (50 mg) combined with serratiopeptidase (15 mg) every eight hours for three days for pain and inflammation control. At the 10-day follow-up visit, uneventful healing with complete epithelialization of the surgical site was observed (Figure 4, Panel B). The patient reported no discomfort and was subsequently referred back to the Department of Orthodontics for treatment continuation.

## Discussion

Soft-tissue embedding of orthodontic appliances is an uncommon but clinically significant complication that may necessitate surgical intervention and multidisciplinary management. The pathogenesis is multifactorial and may involve appliance design, placement technique, tissue characteristics, oral hygiene status, and patient compliance with follow-up visits. TADs placed in non-keratinized mucosa are particularly susceptible to soft-tissue overgrowth because of the reduced resistance of alveolar mucosa to mechanical irritation and tissue remodeling. In addition, excessive insertion depth and inadequate monitoring may predispose mini-implants to progressive mucosal coverage and eventual soft-tissue embedding [8-10].

Orthodontic mini-implants are commonly classified according to their design (cylindrical or tapered), dimensions, insertion method (self-drilling or self-tapping), and placement site, including interradicular and extra-alveolar locations. The choice of mini-implant should be individualized according to the available bone, soft-tissue characteristics, and the intended orthodontic mechanics [11]. Primary stability is a major factor influencing treatment success and depends on factors such as cortical bone quality, implant size, insertion technique, and maintenance of good oral hygiene.

In the first case, complete soft-tissue overgrowth around the orthodontic mini-implant led to pain and functional discomfort. A mini-implant measuring 1.4 mm in diameter and 8 mm in length had been placed in the attached gingiva adjacent to the maxillary second premolar. Although orthodontic mini-implants are associated with high clinical success rates, biological and mechanical complications may occur during treatment. Common complications include peri-implant soft-tissue inflammation, infection, implant loosening, root contact, and screw fracture, whereas injury to adjacent anatomical structures is relatively uncommon. Gingival overgrowth or soft-tissue embedding is a less frequent complication and is often associated with inadequate transmucosal collar height, prolonged treatment duration, plaque accumulation, poor oral hygiene, or placement in non-keratinized mucosa [1,2,8]. In the present case, continuous mechanical irritation combined with plaque retention likely contributed to the inflammatory response and progressive soft-tissue coverage of the implant. Surgical exposure followed by thorough debridement restored access to the mini-implant while preserving the surrounding tissues, and primary wound closure resulted in satisfactory healing with complete resolution of symptoms [12-14].

Orthodontic mini-implants have a high success rate (80-90%); however, complications occur in approximately 13-30% of cases. Soft-tissue inflammation is the most common biological complication (5-22%), while gingival overgrowth and mucosal embedding are reported in 2-15% of patients. These complications are often associated with poor oral hygiene, placement in non-keratinized mucosa, increased soft-tissue thickness, and prolonged implant use [15,16].

The second case involved partial embedding of the TPA loop within the palatal mucosa. The TPA is commonly used to reinforce maxillary anchorage, maintain transverse arch width, correct molar rotation, and stabilize maxillary molars during orthodontic treatment. Although it is a simple and effective appliance, prolonged intraoral use may be associated with complications such as palatal mucosal irritation, plaque accumulation, tissue overgrowth, appliance distortion, and, rarely, soft-tissue embedding. These complications are more likely when the appliance is positioned too close to the palatal mucosa, oral hygiene is inadequate, or regular follow-up is lacking [17,18]. In the present case, continuous mechanical irritation from the closely adapted TPA loop likely promoted localized inflammation and gradual mucosal overgrowth, resulting in partial embedment of the appliance. Surgical retrieval was performed with minimal tissue trauma, and healing by secondary intention was uneventful.

Various techniques have been described for the management of embedded TPA appliances, including conventional surgical exposure and soft-tissue laser-assisted retrieval. Regardless of the method employed, prevention remains the cornerstone of management. Appropriate appliance design, including maintaining adequate clearance between the TPA and palatal tissues, is essential to minimize tissue impingement [18]. Additional modifications, such as repositioning the palatal loop or incorporating a protective sleeve over the loop, have been proposed to reduce chronic mucosal irritation and decrease the likelihood of soft-tissue overgrowth. These preventive measures may contribute to improved patient comfort and reduce the risk of appliance embedment during orthodontic treatment [19].

Both cases highlight the important role of the periodontist in the management of orthodontic complications involving soft tissues. Surgical exposure or retrieval of soft tissue-embedded appliances requires careful tissue handling, effective debridement, and appropriate postoperative care to achieve predictable healing outcomes [13,14]. Furthermore, these cases emphasize the importance of regular clinical monitoring, patient education regarding oral hygiene maintenance, and timely intervention when early signs of tissue impingement or overgrowth are detected.

## Conclusions

The present cases demonstrate that embedding of orthodontic appliances, including mini-implants and TPAs, is a preventable complication that can adversely affect patient comfort and orthodontic treatment progress. Appropriate appliance design, careful placement, maintenance of oral hygiene, and regular follow-up examinations are critical for prevention. When embedding occurs, timely surgical management enables successful retrieval with minimal morbidity and favorable healing outcomes. These cases further underscore the value of close collaboration between orthodontists and periodontists in ensuring the safe and effective management of appliance-related soft-tissue complications.
